# O-56 - INFECTION PREVENTION AND CONTROL IN COMMUNITY SETTINGS: NATIONAL INSIGHTS FROM ACROSS THE HEALTH PROTECTION SYSTEM IN ENGLAND

**DOI:** 10.1093/pubmed/fdag046.050

**Published:** 2026-07-09

**Authors:** Dr Enrique Castro-Sanchez, Ms Stephanie Davis, Ms Shwetha Wevita, Dr Joanna Harris, Dr Jude Robinson

**Affiliations:** UK Health Security Agency; Imperial College London; UK Health Security Agency; UK Health Security Agency; UK Health Security Agency; UK Health Security Agency

## Abstract

**Background:**

Community infection prevention and control (IPC) in England is delivered through a complex system involving UKHSA Health Protection Teams (HPTs), local authorities, NHS partners, and contracted providers, with variation across community and non-NHS settings.

**Objectives:**

To inform a national IPC education and capability-building strategy, we analysed findings from the Future of the Health Protection System (FHPS) Co-Design Group survey to identify challenges and opportunities for improvement.

**Methods:**

Open-ended responses from 192 respondents across the English health protection system were collected between August and October 2024. Reflexive thematic analysis followed data cleaning, using inductive coding with attention to education and governance, supported by NVivo (v12.7). Regional and professional identifiers were retained, and four regional sense-making sessions triangulated findings.

**Results:**

Four interconnected themes were identified. First, respondents reported lack of clarity about responsibility for IPC advice in care homes, schools, and other non-NHS settings. Second, confusion persisted around escalation pathways and leadership during outbreaks, particularly in complex or multi-agency incidents. Third, poor communication and coordination across organisations led to inconsistent messaging. Fourth, limited IPC education infrastructure was described for public health and environmental health professionals.

Respondents also identified strengths. Some regions demonstrated effective partnerships between IPC teams, local authorities, and HPTs, supporting coordinated responses. There was also willingness among staff to engage with flexible educational approaches, including digital platforms and case-based learning.

**Conclusions:**

The findings show system variability that constrains equitable, timely, and preventive IPC delivery in community settings. Clearer roles, stronger governance, improved interoperability, and sustainable funding are needed to enhance preparedness and workforce confidence. The results directly inform a national IPC education strategy aligned with UKHSA priorities and HPT needs.

Next steps include a national self-assessment of IPC education needs and co-creation of tiered training with HPTs, covering statutory knowledge, operational capability, and inter-professional collaboration.

